# Molecular and Technological Characterization of *Saccharomyces cerevisiae* Strains Isolated from Natural Fermentation of Susumaniello Grape Must in Apulia, Southern Italy

**DOI:** 10.1155/2014/897428

**Published:** 2014-01-09

**Authors:** Mariana Tristezza, Lorenagostina Fantastico, Cosimo Vetrano, Gianluca Bleve, Daniela Corallo, Francesco Grieco, Giovanni Mita, Francesco Grieco

**Affiliations:** ^1^C.N.R.-Istituto di Scienze delle Produzioni Alimentari, Unità Operativa di Lecce, Via Provinciale Lecce-Monteroni, 73100 Lecce, Italy; ^2^C.N.R.-Istituto di Scienze delle Produzioni Alimentari, Via Amendola 166/O, 70126 Bari, Italy

## Abstract

The characterization of autochthonous *Saccharomyces cerevisiae* strains is an important step towards the conservation and employment of microbial biodiversity. The utilization of selected autochthonous yeast strains would be a powerful tool to enhance the organoleptic and sensory properties of typical regional wines. In fact, indigenous yeasts are better tailored to a particular must and because of this they are able to praise the peculiarities of the derived wine. The present study described the biodiversity of indigenous *S. cerevisiae* strains isolated from natural must fermentations of an ancient and recently rediscovered Apulian grape cultivar, denoted as “Susumaniello.” The yeast strains denoted by the best oenological and technological features were identified and their fermentative performances were tested by either laboratory assay. Five yeast strains showed that they could be excellent candidates for the production of industrial starter cultures, since they dominated the fermentation process and produced wines characterized by peculiar oenological and organoleptic features.

## 1. Introduction

The conversion of grape must into wine is a fermentative process performed by indigenous microorganisms, whose composition notably contributes to the sensorial and chemical properties of the wine. The main role of yeast during must fermentation is to promote the conversion of grape sugars, in particular hexoses, into ethanol, carbon dioxide, and other metabolites without the production of off-flavours.

In the spontaneous grape must fermentation indigenous yeasts dynamics occurs, it being the final step always dominated by alcohol-tolerant strains [1]. The dominant yeast species is *Saccharomyces cerevisiae* and it was demonstrated that the fermentation process is led and completed by a limited number of strains [2]. Moreover, a sequential substitution of strains happens during must fermentation as they progress to higher ethanol concentration [3].

As the importance of *S. cerevisiae* role in winemaking has long been established, the use of the commercial strains of these yeast cultures in fermentation is an ordinary practice in order to ensure a reproducible product and to reduce the risk of wine spoilage. However, this custom can cause a progressive substitution of local microflora and a consequent reduction of microbial biodiversity. Indeed, knowledge of the autochthonous yeast strains will help to preserve and employ the most representative strains.

The selection and the employment of autochthonous microorganisms could be a powerful instrument to improve the organoleptic and sensory characteristics of wine produced from indigenous grape cultivars [4]. In fact, autochthonous yeasts are the microorganisms better adapted to a specific must, which detain characteristics determined by the variety of the grapes and the terroir and therefore they are able to exalt the peculiarities (aromas, structure, and colour) of a wine [5].

Apulia is the second Italian wine-producing region and its wine industry is at the present living a moment of large qualitative evolution, by implementing the use of innovative systems to guarantee and praise the peculiar properties of regional wines. Therefore, there is an increasing demand by local wine producers for selecting, among the indigenous yeast flora, autochthonous strains with peculiar oenological features which could be considered representative of an oenological region [6]. To date, several investigations have described the enological performances of *S. cerevisiae* strains isolated from red and white grapes in Southern Italy [7] and, in particular, from Aglianico [8], Nero d'Avola [9], and Negroamaro [10] musts. The aim of this study was to investigate for the first time the molecular and technological characterization of indigenous *S. cerevisiae* strains isolated form natural fermentation of must obtained from Susumaniello grapes.

The Susumaniello is an ancient red variety only grown in Salento (Southern Italy), recently rediscovered and revived in the attention of the market. The bouquet of the obtained wines is peculiar since its aromas range from fruity notes like cherries to notes of freshly mown grass, with a fruity finish with added notes of flowers. The employment of selected autochthonous yeast strains would be a powerful tool to enhance the organoleptic and sensory properties of this typical regional wine and to better tie the Susumaniello wine to its terroir.

As a first step, we proceeded to the molecular characterization of a representative yeast population isolated at the end of the alcoholic fermentation process. Then, we evaluate the oenological properties and technological properties of different strains of *S. cerevisiae* identified by molecular analysis. This study represents the starting point of a clonal selection of yeasts for grape from Susumaniello, addressed to the characterization of indigenous strains denoted by excellent enological properties, to be used in the future for the preparation of native fermentation starter.

## 2. Materials and Methods

### 2.1. Natural Fermentation and Must Sampling

Sampling from spontaneous fermented must was conducted according to Grieco et al. [6]. Briefly, grapes (*Vitis vinifera*) were harvested in Cellino San Marco (Brindisi), which is one of the most important areas for Susumaniello cultivar production in Apulia, during the 2011 vintage. The spontaneous fermentation was carried out in a newly established experimental cellar. The grapes were smashed and the obtained must (sugars 230 g/L, 19.9° Ba, pH 3.37, assimilable nitrogen concentration 168.5 g/L) was added with 100 mg/L potassium metabisulphite. The fermentation was performed in a stainless steel 100 L tank that was previously sterilized by treatment with 5% sodium hypochlorite followed by extensive washing with tap water. The fermentation process was monitored by measuring the residual sugar. Must and residual lees were sampled at the end of alcoholic fermentation (0-1° Ba), combined with 20% sterile glycerol and stored at −80°C until analysis.

### 2.2. Yeast Isolates Selection and Molecular Characterization

Yeast isolates were firstly screened for their ability to produce hydrogen sulphide on Biggy agar (Sigma, USA) as described by Tristezza et al. [10]. At least 200 H_2_S-low producer isolates (i.e., white or light brown colonies) were selected for further characterization. Thus, 72 colonies were randomly selected and yeast total genomic DNA was prepared according to Tristezza et al. [11]. The isolates were identified according to the length of the rDNA region spanning the 5.8 S rRNA gene and flanking the internal transcribed spacers 1 and 2 by using the primers ITS1 (5′-TCCGTAGGTGAACCTGCGG-3′) and ITS4 (5′-TCCTCCGCTTATTGATATGC-3′) [12]. The PCR conditions were those described by Cappello et al. [13]. To determine the species identity of isolates, amplicons were subjected to sequence analysis and compared with the sequences in the GenBank database [14]. *S. cerevisiae* isolates were characterized at strain level by interdelta typing according to Legras and Karst and employing the primers delta12 (5′-TCAACAATGGAATCCCAAC-3′) and delta21 (5′-CATCTTAACACCGTATATGA-3′). [15]. Amplification products were separated by gel electrophoresis as previously described [14].

### 2.3. Microvinifications

The identified strains were tested by a microfermentation assay in Susumaniello grapes must (sugars 226 g/L, 20.5° Ba, pH 3.35) sterilized as described by Grieco et al. [6]. One hundred millilitres of must was placed in sterile 150 mL flasks and then inoculated with 10^6^ CFU/mL of yeast inoculum precultured in the same must. The samples were incubated at 25°C and weighted daily to follow the weight loss caused by CO_2_ production until constant weight. The samples of fermented must were stored at −20°C until required for analysis. Each fermentation experiment was carried out by performing three simultaneous independent repetitions. The commercial starter CM was used as control since it was the most used strain by the winemakers in the sampled area.

### 2.4. Analytical Determinations

Hydrogen sulphide production was detected by the blackening of the PbAcO strip [16]. Fermentation rate (FR), fermentation purity (FP), and alcohol yield coefficient (AYC) were calculated according to Tristezza et al. [10]. Wines and musts were analysed by Fourier transform infrared spectroscopy (FTIR), employing the WineScan Flex (FOSS Analytical, Denmark). Samples were centrifuged at 8,000 rpm for 10 min and then analysed following the supplier's instructions. The major volatile constituents (acetaldehyde, ethyl acetate, 2-methyl-1-propanol, higher alcohols (3-methyl- and 2-methyl-1-butanol), and acetoin) were determined by gas chromatography as previously described [17].

### 2.5. Statistical Treatment of Data

Agarose gels were scanned with a Gel Doc 1000 apparatus (Bio-Rad, USA) and analyzed with Molecular Analyst software (Bio-Rad, USA). The obtained data matrix was employed for cluster analysis by UPGMA algorithm using the Dice coefficient. Microbial diversity indices, such as the richness (S) of the *S. cerevisiae* community, the Shannon index of general diversity (H), Simpson's index of diversity (1-D), the Evenness (e^∧^H/S), Berger-Parker dominance, and variability were calculated according to Tristezza et al. [18].

Significant differences among selected strains were determined for each chemical and volatile compound based one-way ANOVA; Tukey HSD post hoc tests were applied to establish significant differences between means (*α* = 0.05). The contribution of these variables to the differences between strains was estimated by principal component analysis (PCA).

Cluster analysis, microbial diversity indices calculation, and PCA were performed using the free software package PAST [19]. The analysis of variance was performed using the SigmaStat software version 11.0 (Systat Software Inc., Chicago, IL, USA).

## 3. Results

**Figure 1:**
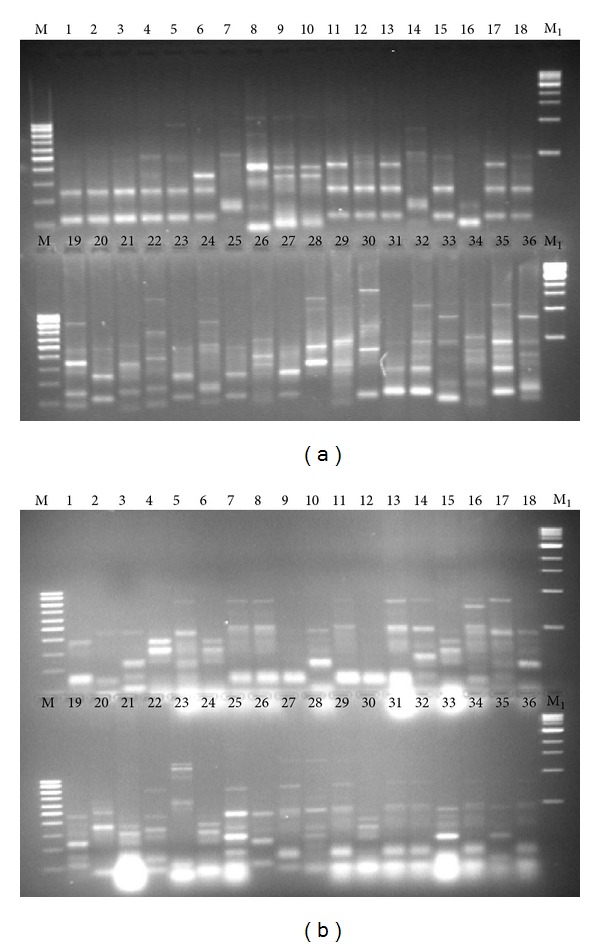
Electrophoretic patterns of interdelta region obtained from seventy-two *Saccharomyces cerevisiae* randomly isolated at the end of spontaneous fermentation of Susumaniello grapes. Panel (a): lines 1 to 36 correspond to strains from S1 to S36. Panel (b): lines 1 to 36 correspond to strains from S37 to S72. M, 100 bp DNA Ladder (New England Biolabs, USA); M_1_, 1 Kb DNA Ladder (New England Biolabs, USA).

**Figure 2:**
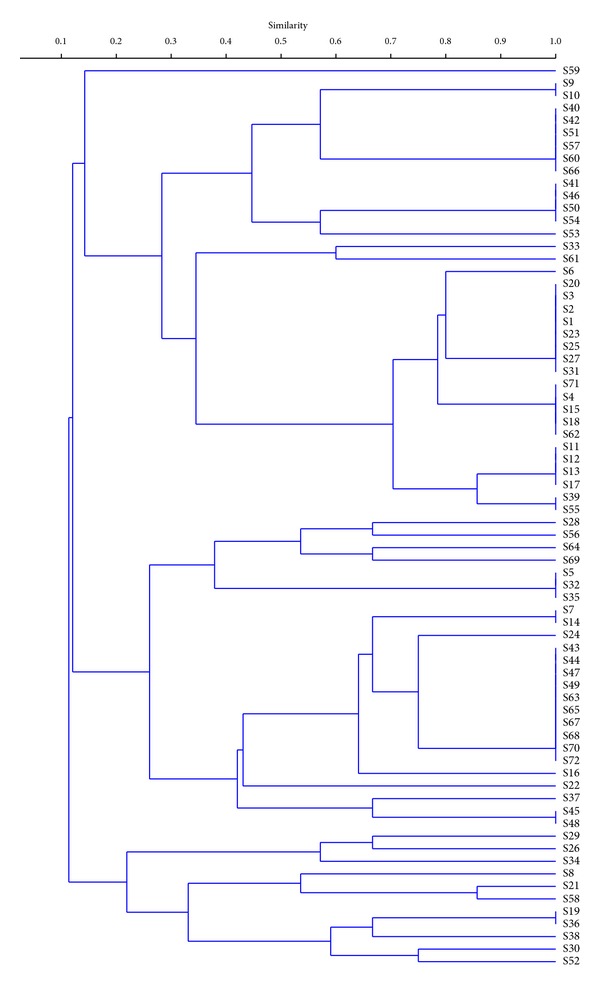
UPGMA dendrogram generated by cluster analysis of interdelta region patterns obtained from the *Saccharomyces cerevisiae* strains isolated during the later stages of spontaneous fermentation of Susumaniello grapes. Calculated percentages of similarity are given on the axis.

As the first step of the selection of oenological autochthonous yeasts associated with natural fermentations of Susumaniello grapes, serial dilutions of must and lees collected at the end of spontaneous fermentation were spread on selective solid medium, thus allowing the isolation of 208 yeast colonies no or low H_2_S producers. The above 208 isolates were identified by molecular analysis of yeast rDNA, thus confirming that they all belonged to the species *Saccharomyces cerevisiae*. Seventy-two isolates randomly selected were characterized at strain level using a PCR-based assay, relying on the amplification of interdelta regions (Figure 1). The obtained molecular fingerprinting enabled the clustering of the *S. cerevisiae* population in 34 different strains (Figure 2). The *S. cerevisiae* population not only showed an elevated polymorphism, calculated as the ratio between the number of molecular patterns and the number of isolates (47%), but the total number of individuals in the sample was quite evenly distributed between the strains (evenness index 0.70); thus the proportional abundance of the most abundant type was quite low (Berger-Parker index, 0.14), which gave rise to higher indices of general biodiversity (*H* > 3) and low concentration of dominance (*D* = 0.06) (Table 1).

All the identified strains were deposited in the ISPA Collection (http://server.ispa.cnr.it/ITEM/Collection/) and then they were tested in microfermentation assays, in order to evaluate strain-specific technological and oenological properties (Table 2). We firstly evaluated the data obtained from the microfermentation assay for three major descriptors: acetic acid concentration (<0.6 g/L); total sugar consumption (<4 g/L); no H_2_S production during fermentation (<8 mg/L). Six strains (S19, S26, S28, S29, S33, and S38) produced concentration of acetic acid over 0.6 g/L (data not shown), as indicated by their very high values of FP (fermentation purity) indices (Table 2). The wines produced with strains S1, S22, S30, S34, S37, S53, S58, S59, and S64 showed a presence of residual sugar in the must >4 g/L (data not shown) demonstrating low alcohol yield coefficients (AYCs; <0.5). Furthermore, isolates S24, S48, S56, S61, and S69 produced hydrogen sulphide during fermentation. This primary screening indicated that fifteen indigenous yeast strains (S5, S6, S7, S8, S9, S12, S16, S21, S24, S39, S40, S41, S47, 52, and S71) satisfied the cross-evaluation of the chosen major descriptors in microfermentation assays. These strains were selected to be further characterized. The main chemical and volatile compounds present in musts fermented by each one of them are shown in Tables 3 and 4, respectively. The production of ethanol was proportional to the consumption of sugars, higher for the strains S8, S71, S39, and S40 (resp. 12.60%, 12.53%, 12.24%, and 12.16%). The fifteen strains have produced satisfactory amount of glycerol, with values of up to 7.21 g/L (Table 3). The quantitative analysis of other organic acids revealed that malic acid (ranging from 2.45 g/L to 3.00 g/L) and tartaric acid (with a range of values between 2.07 g/L and 3.40 g/L) were the most abundant, while little amounts of lactic acid were observed only in three samples (S7, S24, S47, and S52).

The strain-specific aptitude to produce volatile compounds involved in the wine flavour was also evaluated and the results are shown in Table 4. The acetaldehyde is one of the most important carbonyl compounds produced during fermentation; at low levels it contributes to fruity flavour, while high concentrations (>200 mg/L) confer flatness to wines. All strains of *S. cerevisiae* produced a quantity of this compound, within the range between 5.0 mg/L (strain S39) and 23.05 mg/L (strain S16). Ethyl acetate may contribute to the wine aroma with pleasant, fruity fragrance if present at concentrations lower than 150 mg/L, whereas, at higher concentrations, it produces a sour-vinegar off-odour. In our experiments, the ethyl acetate was detected in quantities ranging from 23.49 mg/L (strain S24) to 61.47 mg/L (strain S8). As regards the production of higher alcohols during fermentation, the amounts of 2-methyl-1-propanol (isobutyl alcohol) detected ranged from 13.26 mg/L (strain S47) to 49.70 mg/L (strain S52) and the summation of amyl alcohols ranged from 55.77 mg/L (strain S47) to 138.73 mg/L (strain S16). All the strains under study, except for strain S21, produced little quantity of acetoin (3-hydroxy-2-butanone) under its odour threshold (150 mg/L).

**Figure 3:**
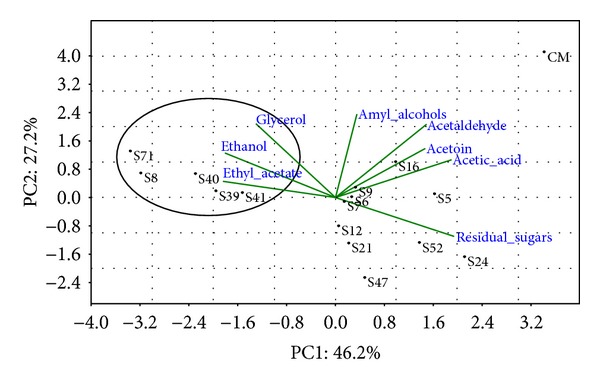
Principal component analysis (PCA) performed employing as variables the data obtained by the chemical analysis of must fermented with the selected strains. The projection on the plan of the different variables is indicated in the plot by lines.

The principal component Analysis (PCA) was applied to the matrix of multivariate data comprising concentrations of eight compounds (acetic acid, acetoin, ethyl acetate, amyl alcohols, acetaldehyde, residual sugars, ethanol, and glycerol) for the fifteen strains under study (Figure 3). Data concerning the hydrogen sulphide production (Table 2) were not included in the PCA, since they were not continuous. Along the first component, the samples were clearly grouped in three clusters. Samples S71, S8, S40, S39, and S41 were grouped in a cluster on the left part of the plot, whereas the other samples (S5, S6, S7, S9, S12, S16, S21, S24, S47, and S52) were grouped into a more scattered cluster on the right side of the plot. The third cluster on PC1 only included the commercial strain. The loadings of each compound on the principal components show that ethyl acetate, ethanol, and glycerol are mainly responsible for the first cluster, whereas the residual sugars characterize the samples S12, S21, S24, S47, and S52 and acetic acid and acetoin and acetaldehyde are responsible for the differentiation of the strains S5, S6, S7, and S16.

## 4. Discussion

The present study was aimed at the molecular and technological characterization of *Saccharomyces cerevisiae* strains isolated during the process of natural fermentation of must from the Susumaniello grape cultivar in Apulia (Southern Italy), in order to select suitable autochthonous starter cultures for the improvement of oenological production of this typical regional wines. Indeed, the Susumaniello grape cultivar has been recently rediscovered by the regional wine industry, thanks to the uniqueness and the quality of red wines made from its must.

Numerous examples of microvinification protocols are present in the literature [20, 21], but such procedures have all been developed at the laboratory level and therefore are far to reproduce a natural fermentation process. In fact, preliminary studies performed during the 2006 vintage (Grieco F., personal communication) have shown that natural fermentations carried out from a quantity of grapes <20 kg have very frequently stuck the process of fermentation, maybe due to a not sufficient amount of nutrients and nutritional factors required by the wild yeast cells during the proliferative phase [22, 23] or by the scarce presence of *S. cerevisiae* cells on the grape berries [24]. We have overcome this problem by performing the winemaking process using 100 kg of grapes in a tailor-designed experimental cellar, without contact with any industrial vinification plant. This protocol allowed to reproduce the fermentative process on a significant scale, but maintaining the microbiological control of the process and, given the impossibility of contamination by industrial strains, it has proved to be an excellent tool for the implementation of a protocol for native microflora identification [10].

The must sampling has been carried out at the end of fermentation with the aim of analyzing the population of *S. cerevisiae* yeast present in the most significant phase of fermentation, as shown in numerous studies that addressed the analysis of the microflora in natural fermentation of typical varieties of different geographic origin [2, 25–28]. We did not identify isolates belonging to *non-Saccharomyces* species and this finding is likely due to the absence in the analyzed population of *non-Saccharomyces* strains resistant to high ethanol and SO_2_ concentrations.

The first selection step was performed by choosing the yeast isolates according to their aptitude to not produce hydrogen sulfide during must fermentation, since this compound is highly undesirable because it is perceptible even at minimal concentrations (<8 mg/L) with an unpleasant odor of rotten eggs [29].

Several different genetic fingerprinting techniques, such as karyotype analysis [30], microsatellite genotyping [31], mtDNA restriction analysis [32], and interdelta sequence typing [15] have been successfully applied for genotyping autochthonous wine yeast strains. However, the method adopted in this study, that is, the PCR amplification of interdelta sequences, is the most appropriate method for a large scale application [33].

No genetic relations nor identity was found between the 72 analyzed isolates and the CM strain used as control (Figure  1S). This finding was absolutely expected since the spontaneous fermentation was carried out in a sterile tank and in an experimental cellar far from any industrial cellar.

The population dynamics of *S. cerevisiae* strains revealed interesting aspects from the ecological point of view. The intraspecific variability detected in the population (47%) was high compared with other indigenous populations of *S. cerevisiae* present in musts from France [2, 34], Spain [35, 36], and Argentine [37]. In addition, there wasn't a predominant strain at the end of fermentation (*D* = 0.06). This finding is extremely interesting and differs from the evidence produced by previous studies [35, 38, 39] but is in agreement with others [6, 10, 40] which reported an unusually high number of different strains of *S. cerevisiae* in spontaneous fermentation of grapes. This evidence is explainable by the fact that the experimental vinification system used in this study was not influenced by the microflora present in the cellar.

The strains characterized by molecular analysis were used in microfermentations assays that have allowed us to evaluate their technological and enological properties. Out of the 72 identified strains, 15 were able to efficiently complete the fermentation process. In fact, the strains S5, S6, S7, S8, S9, S12, S16, S21, S24, S39, S40, S41, S47, S52, and S71 have shown

- to efficiently consume the sugars, it being the presence of residual sugar (>4 g/L) indicative of incomplete fermentation [41, 42];
- to be low producers of acetic acid, since the high production (>0.6 g/L) of this organic acid by *S. cerevisiae* is extremely unwanted [43].

The strains S5, S21, and S52 have been those who have almost entirely consumed reducing sugars during alcoholic fermentation yet also have brought lower values of alcohol yield coefficient. This finding allows us to hypothesize that the sugars in these strains are also used by different metabolic pathways, without affecting the purity of the fermentation in general.

The study of the kinetics of fermentation was based on the evaluation of the initial and final speed of fermentation [6]. The initial rate of fermentation is synonymous with a rapid colonizing ability of the yeast during the initial phase of the fermentation [44]. Strains S21, S41, S40, and S8 have demonstrated a higher initial speed, when compared with data obtained by the other eleven strains. The fermentation purity, indicated as the ratio between the volatile acid and the ethyl alcohol formed product, has been one of the parameters evaluated in this work. The values found in the samples tested are all below the maximum value (0.12) indicated for *S. cerevisiae* in similar previous studies [45].

The glycerol produced by yeast during the must fermentation is one of the major components of the wine, where usually it is found in a concentration ranging from 5 to 8 g/L [46, 47]. Glycerol has a fundamental role in the formation of the bouquet of the wine, as it contributes to improving the balance and the body [46]. The S41 and S71 strains were the major glycerol producers of values ≥7 g/L.

The selected yeast strains confirmed their unability to produce sulfur dioxide. The initial concentration of sulfates influences the production of SO_2_ and H_2_S, as shown by a study that highlighted various aspects regarding the interaction of *S. cerevisiae* with sulfur compounds [48].

Numerous compounds, produced by yeast during the fermentation process, determine the organoleptic quality of the final wine [29, 49–53]. The level of these substances allows to differentiate at the strain level different yeasts species [54]. In order to determine the possible influence of each of the 34 strains isolated in studio in the formation of the aroma of the wine, the gas chromatographic profiles of microfermentations carried were obtained and compared.

It is known that most of the higher alcohols is of fermentative origin, since it has been demonstrated that their production is linked to the microbial metabolism of amino acids [52]. The higher alcohols are present in wines in total concentrations ranging from 150 to 550 mg/L [55]. The concentrations of 2-methyl-1-propanol (isobutyl alcohol) and the summation of 2-methyl-1-butanol and 3-methyl-1-butanol have been the subject of these analyses, since these compounds have an important role in determining the wine bouquet.

The 15 *S. cerevisiae* indigenous selected strains have produced concentrations of these compounds lower than 300 mg/L, indicating that all strains participate in the positive aromatic complexity of the wine. In fact, the acetates of higher alcohols give, in concentrations of less than 300 mg/L, special fruity and floral notes, while at higher concentrations they negatively affect the wine flavor [52].

The ethyl acetate is one of the most important ester of wine and it is formed by the yeast during fermentation as strain-specific feature [52, 56]. The ethyl acetate in concentrations ranging between 50 and 80 mg/L positively contributes to the aroma of the wine base [57], while when the concentration exceeds the threshold of olfactory perception (160 mg/L), it contributes negatively to bouquet of the wine, as it introduces a strong acetic acid note. The fifteen strains produced ethyl acetate in concentration lower than 160 mg/L, thus showing that they could positively contribute to the aroma composition of wines produced.

The possible contribution of the 15 analyzed strains of *S. cerevisiae* in the formation of the aroma of the wines was analyzed through the evaluation of a parameter that is obtained from the relationship between foreign and amyl alcohols (E/A, [58]). The E/A values ≥0.5 were obtained for strains S8, S39, S40, S47, S52, and S71, confirming that these strains can potentially enrich the aromatic composition of Susumaniello wines.

The acetaldehyde, the predominant aldehyde in the wine, is a product of the alcoholic fermentation and its content in wine varies from 10 to 300 mg/L. When this compound is present in wine in the above range, it gives a pleasant aroma of fruit, but at higher concentrations its presence is perceived as an unpleasant pungent odor. The strains under study have proven to be all low producers of acetaldehyde; in fact concentration values of the compound less than 30 mg/L were obtained.

It is important to underline that the principal component analysis (PCA) applied the data matrix constituted by concentrations of the most important chemical parameters for the analyzed identified strains allowing their clear-cut differentiation form the commercial strain used as control, which showed the poorest enological performances.

The results of the PCA confirmed the evidence given by the analytical assays, making it possible to conclude that, among all the indigenous strains of *S. cerevisiae* analyzed, the strains S8, S39, S40, S41, and S71 resulted that the strain was endowed with the best enological properties.

## 5. Conclusions

The result of principal component analysis confirmed the evidence given by the analytical assays, making it possible to conclude that, among all the 34 indigenous strains of *S. cerevisiae* analyzed, the strains S8, S39, S40, S41, and S71 detain the enological and technological properties that suggest them as promising candidate for the production of a fermentation starter for the industrial production of Susumaniello wine, as powerful instrument to improve the organoleptic and sensory characteristics of the product. In conclusion, this work represents the first phase of a wider project for the qualitative improvement of Susumaniello wine, which will industrially employ indigenous fermentative starters as possible strategy to enhance the organoleptic complexity of this autochthonous grape cultivar and to tie Susumaniello wine to the culture and history of the production area.

## Supplementary Material

FIG. 1S – Electrophoretic patterns of interdelta region obtained from the CM (lane 1) and an unrelated S. cerevisiae (lane 2) strains. M1, 1Kb DNA Ladder (New England Biolabs, USA).

## Figures and Tables

**Table 1:** Intraspecific diversity indices of *S. cerevisiae* population calculated from cluster analysis.

No. of *S. cerevisiae* isolates	72
No. of different molecular patterns (S)	34
Variability (%)^a^	47
Simpson index (1-D)	0.94
Shannon index (H)	3.17
Evenness (e^*∧*^H/S)	0.70
Berger-Parker dominance	0.14

^a^Ratio between the number of patterns and the number of isolates.

**Table 2:** Main oenological and technological properties determined in thirty-four autochthonous *S. cerevisiae *strains and one commercial starter (CM).

Isolate	ITEM no.	FR	FP	AYC	H_2_S^a^
S1	9497	1.7	0.04	0.41	+
S5	9499	1.9	0.03	0.49	−
S6	9500	2.4	0.03	0.52	−
S7	9501	2.4	0.02	0.53	−
S8	9502	3.5	0.01	0.56	−
S9	9503	2.7	0.03	0.53	−
S12	9504	2.9	0.02	0.52	−
S16	9505	3.2	0.03	0.51	−
S19	9506	2.2	0.70	0.39	−
S21	9507	4.2	0.02	0.48	−
S22	9508	1.8	0.05	0.26	+
S24	9509	2.2	0.03	0.50	−
S26	9510	2.50	0.94	0.30	−
S28	9511	2.21	0.49	0.38	−
S29	9512	2.18	0.23	0.34	−
S30	9513	2.48	0.03	0.43	−
S33	9514	2.65	0.67	0.33	−
S34	9515	1.86	0.05	0.28	−
S37	9516	2.50	0.04	0.36	−
S38	9517	1.78	0.12	0.36	−
S39	9518	3.0	0.02	0.55	−
S40	9519	3.6	0.02	0.54	−
S41	9520	3.9	0.02	0.52	−
S47	9521	2.2	0.03	0.49	−
S48	9522	3.2	0.03	0.47	+
S52	9523	1.3	0.02	0.47	−
S53	9524	2.20	0.04	0.27	+
S56	9525	2.50	0.04	0.49	+
S58	9526	1.78	0.04	0.43	+
S59	9527	1.10	0.07	0.29	−
S61	9528	2.41	0.04	0.47	+
S64	9529	2.30	0.05	0.29	+
S69	9530	3.00	0.03	0.51	+
S71	9531	2.7	0.02	0.56	−
CM	—	4.7	0.04	0.50	+

Data, measured at the end of fermentation, represent the average of three replicates. ITEM: ISPA's agrofood toxigenic fungi culture collection.

FP: fermentation purity (volatile acidity (g/L)/ethanol (% v/v)), AYC: alcohol yield coefficient (alcohol (% v/v)/(initial sugars (%)−residual sugars (%))), FR: fermentation rate (g of CO_2_/day, after 3 days of fermentation). ^a^H_2_S production: absent (−); low (+), high (++), and very high (+++).

**Table 3:** Concentration of major chemical compounds in fermented musts obtained with fifteen autochthonous *S. cerevisiae* and one commercial starter (CM).

ID	Ethanol (%v/v)	Sugars (g/L)	Total acidity (g/L)	Volatile acidity (g/L)	pH	Malic acid (g/L)	Lactic acid (g/L)	Tartaric acid (g/L)	Glycerol (g/L)	Polyphenols (mg/L)
S5	10.97 ± 0.00^a,b^	2.70 ± 0.05^a,b^	5.06 ± 0.01^a^	0.37 ± 0.01^a,b^	3.33 ± 0.01^a,b^	2.94 ± 0.01^a,b^	n.d.	2.69 ± 0.00^b,c^	3.92 ± 0.06^a,d^	1075 ± 7^e,f,g^
S6	11.59 ± 0.23^a,b^	2.74 ± 0.01^a,b^	5.97 ± 0.26^a^	0.31 ± 0.01^b^	3.29 ± 0.00^a,b^	2.89 ± 0.11^a,b^	n.d.	3.20 ± 0.12^a,b^	5.52 ± 0.20^a,c^	1615 ± 58^c,d^
S7	11.93 ± 0.00^a,b^	2.35 ± 0.04^a,b^	6.40 ± 0.01^a^	0.26 ± 0.01^b,c^	3.41 ± 0.00^a,b^	2.78 ± 0.01^a,b^	0.18 ± 0.02^b^	2.81 ± 0.02^b^	3.68 ± 0.07^b,c,d^	1120 ± 0.1^e,f^
S8	12.60 ± 0.01^a^	1.98 ± 0.08^a,b^	6.59 ± 0.01^a^	0.14 ± 0.01^c^	3.22 ± 0.01^a^	2.87 ± 0.00^a,b^	n.d.	3.40 ± 0.03^a^	5.81 ± 0.11^a,b^	1749 ± 14^b,c^
S9	11.84 ± 0.00^a,b^	2.57 ± 0.11^a,b^	5.97 ± 0.01^a^	0.32 ± 0.01^b^	3.33 ± 0.00^a,b^	2.93 ± 0.06^a,b^	n.d.	2.77 ± 0.05^b^	4.15 ± 0.04^a,c^	1150 ± 25^e^
S12	11.58 ± 0.12^a,b^	2.64 ± 0.07^a,b^	5.95 ± 0.07^a^	0.27 ± 0.01^b^	3.34 ± 0.01^a,b^	2.99 ± 0.03^a^	n.d.	2.75 ± 0.03^b^	4.20 ± 0.08^a,c^	1212 ± 10^e^
S16	11.34 ± 0.07^a,b^	2.67 ± 0.00^a,b^	5.68 ± 0.04^a^	0.31 ± 0.01^b^	3.32 ± 0.00^a,b^	2.89 ± 0.02^a,b^	n.d.	3.09 ± 0.03^a,b^	5.14 ± 0.07^a,c^	1501 ± 12^d^
S21	10.65 ± 0.00^a,b^	2.94 ± 0.05^a,b^	5.32 ± 0.01^a^	0.24 ± 0.01^b,c^	3.30 ± 0.10^a,b^	2.82 ± 0.30^a,b^	n.d.	3.08 ± 0.03^a,b^	4.58 ± 0.21^a,c^	1250 ± 10^e^
S24	11.04 ± 0.00^a,b^	3.15 ± 0.00^a^	5.82 ± 0.02^a^	0.34 ± 0.00^b^	3.41 ± 0.00^a,b^	2.68 ± 0.02^a,b^	0.29 ± 0.06^b^	2.38 ± 0.01^c^	3.15 ± 0.01^c,d^	932 ± 23^g^
S39	12.24 ± 0.04^a^	2.25 ± 0.03^a,b^	6.68 ± 0.01^a^	0.24 ± 0.00^b,c^	3.28 ± 0.00^a^	2.92 ± 0.00^a,b^	n.d.	3.18 ± 0.03^a,b^	6.84 ± 0.07^a^	1765 ± 4^b,c^
S40	12.16 ± 0.00^a^	2.18 ± 0.00^a,b^	6.65 ± 0.67^a^	0.26 ± 0.01^b,c^	3.32 ± 0.01^a,b^	2.86 ± 0.00^a,b^	n.d.	2.07 ± 0.01^d^	6.51 ± 0.25^a^	1763 ± 36^b,c^
S41	11.51 ± 0.03^a,b^	2.65 ± 0.05^a,b^	6.94 ± 0.07^a^	0.21 ± 0.01^b,c^	3.27 ± 0.01^a^	3.00 ± 0.01^a^	n.d.	3.32 ± 0.03^a^	7.02 ± 0.01^a^	1841 ± 17^b^
S47	10.88 ± 0.00^a,b^	3.00 ± 0.00^a^	6.25 ± 0.01^a^	0.30 ± 0.01^b^	3.37 ± 0.01^a,b^	2.75 ± 0.00^a,b^	0.25 ± 0.00^b^	2.35 ± 0.00^c^	3.55 ± 0.00^c,d^	990 ± 6^f,g^
S52	10.46 ± 0.72^b^	2.82 ± 0.78^a,b^	6.77 ± 0.40^a^	0.23 ± 0.01^b,c^	3.41 ± 0.02^b^	2.45 ± 0.16^b^	0.43 ± 0.05^a^	2.27 ± 0.12^c,d^	2.18 ± 0.60^d^	770 ± 34^h^
S71	12.53 ± 0.05^a^	1.47 ± 0.01^b^	6.84 ± 0.01^a^	0.23 ± 0.02^b,c^	3.29 ± 0.00^a^	2.97 ± 0.05^a^	n.d.	3.22 ± 0.03^a,b^	7.21 ± 0.00^a^	1943 ± 5^a^
CM	11.23 ± 0.24^a,b^	2.90 ± 0.17^a,b^	6.61 ± 0.11^a^	0.46 ± 0.02^a^	3.33 ± 0.02^a,b^	2.88 ± 0.06^a,b^	n.d.	2.95 ± 0.01^bs^	6.69 ± 0.39^a^	1872 ± 23^a,b^

Values are the mean of two injections of each replicate (*n* = 6); the standard deviation values (±) are indicated. Different letters indicate means that differ significantly (*α* = 0.05). n.d.: not detectable.

**Table 4:** Concentration of major volatile compounds in fermented musts obtained with fifteen autochthonous and one commercial (CM) strain of *S. cerevisiae*.

Isolate	Acetaldehyde	Ethyl acetate	2-Methyl-1-propanol	Amyl alcohols	Acetoin	E/A
S5	12.93 ± 1.3^b,c,d^	40.06 ± 2.9^a,b^	35.63 ± 0.8^b,d^	114.41 ± 0.45^a,d,e^	6.80 ± 0.6^a,b^	0.35
S6	9.30 ± 0.5^b,c,d^	27.16 ± 6.3^a,b^	31.74 ± 0.5^c,d^	128.18 ± 0.5^f,d^	2.23 ± 2.2^a^	0.21
S7	19.78 ± 0.8^b,c^	27.01 ± 0.6^b,c^	32.32 ± 0.7^c,d^	111.94 ± 0.75^a,d^	4.07 ± 0.5^a^	0.24
S8	5.40 ± 0.2^c,d^	61.47 ± 3.1^a^	26.87 ± 1.3^d^	124.88 ± 2.0^d,e^	3.42 ± 0.4^a^	0.49
S9	22.15 ± 4.0^b^	37.67 ± 2.6^a,b^	36.60 ± 1.2^b,c^	111.05 ± 1.1^a,b,c^	2.92 ± 0.6^a^	0.34
S12	6.64 ± 0.4^c,d^	30.72 ± 1.1^a,b^	28.02 ± 0.8^d^	104.12 ± 1.55^a^	3.10 ± 0.7^a^	0.30
S16	23.05 ± 6.2^b^	34.13 ± 2.5^a,b^	42.01 ± 0.6^b^	138.73 ± 0.9^f^	4.39 ± 0.3^a^	0.25
S21	6.70 ± 0.2^c,d^	33.66 ± 1.2^a,b^	30.81 ± 1.1^c,d^	132.02 ± 0.65^e,f^	n.d.	0.25
S24	16.22 ± 1.4^b,c^	23.49 ± 2.0^b^	17.30 ± 1.1^e,f^	73.68 ± 0.8^g^	4.21 ± 0.7^a^	0.32
S39	5.00 ± 0.1^d^	40.10 ± 2.6^a,b^	29.70 ± 0.3^d^	105.20 ± 1.4^a^	2.20 ± 0.3^a^	0.53
S40	10.10 ± 0.1^b,d^	59.97 ± 4.4^a^	29.55 ± 0.1^d^	114.71 ± 4.15^b,d,e^	1.69 ± 0.1^a^	0.52
S41	5.32 ± 0.3^d^	55.64 ± 4.4^a^	19.23 ± 1.9^e^	127.00 ± 1.65^d,e^	2.18 ± 0.3^a^	0.44
S47	12.30 ± 0.2^b,d^	44.97 ± 6.1^a,c^	13.26 ± 0.9^f^	55.77 ± 1.0^h^	2.00 ± 1.0^a^	0.81
S52	5.80 ± 1.0^c,d^	37.90 ± 1.8^a,b^	49.70 ± 1.3^a^	121.00 ± 1.55^c,d,e^	6.5 ± 0.5^a,b^	0.53
S71	7.42 ± 0.4^c,d^	56.28 ± 5.5^a^	28.97 ± 0.4^d^	108.51 ± 1.2^a,b^	2.62 ± 0.3^a^	0.52
CM	59.65 ± 3.1^a^	30.17 ± 0.6^a,b^	49.18 ± 0.6^a^	173.62 ± 0.6^i^	8.52 ± 0.8^b^	0.17

Values expressed in mg/L are the mean of three injections of each replicate (*n* = 9); the standard deviation values (±) are indicated. Different letters indicate means that differ significantly (*α* = 0.05). E/A: ethyl acetate/amyl alcohols; n.d.: not detectable.
