# Supplementary material for: Molecular and Technological Characterization of Saccharomyces cerevisiae Strains Isolated from Natural Fermentation of Susumaniello Grape Must in Apulia, Southern Italy
Source: Int J Microbiol. 2014 Jan 9;2014:897428. doi: 10.1155/2014/897428 (PMC3942102; doi:10.1155/2014/897428)
Supplement: Supplementary file 1 — FIG. 1S – Electrophoretic patterns of interdelta region obtained from the CM (lane 1) and an unrelated S. cerevisiae (lane 2) strains. M1, 1Kb DNA Ladder (New England Biolabs, USA). [file 897428.f1.pdf]

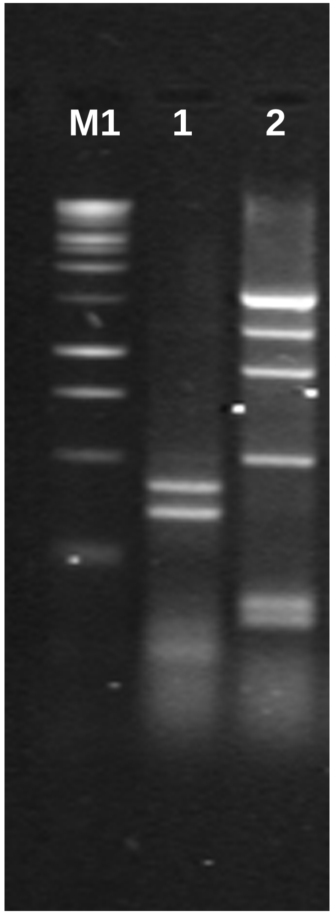

FIG. 1 – Electrophoretic patterns of interdelta region obtained from the CM (lane 1) and an unrelated *S. cerevisiae* (lane 2) strains. M1, 1Kb DNA Ladder (New England Biolabs, USA).
